# Clinical Implications of Acute Stent Mal-Apposition in the Left Main Coronary Artery

**DOI:** 10.31083/j.rcm2506196

**Published:** 2024-05-29

**Authors:** Xi Wu, Mingxing Wu, Haobo Huang, Lei Wang, Zhe Liu, Jie Cai, He Huang

**Affiliations:** ^1^Department of Cardiology, Xiangtan Central Hospital, 411100 Xiangtan, Hunan, China

**Keywords:** drug-eluting stent, mal-apposition, intravascular ultrasound, percutaneous coronary intervention, left main coronary artery

## Abstract

**Background:**

Intravascular ultrasound (IVUS) has been utilized to determine acute stent mal-apposition (ASM) after percutaneous coronary intervention (PCI) in the left main coronary artery (LMCA). However, the clinical consequences of this finding remain uncertain. This research aimed to evaluate the clinical implications of ASM in the LMCA using IVUS.

**Methods:**

In this study, 408 patients who underwent successful drug-eluting stent (DES) implantation in the LMCA were evaluated. We analyzed the prevalence and characteristics of ASM and its correlation with clinical outcomes. ASM is characterized by stent struts that are not in immediate proximity to the intimal surface of the vessel wall after initial stent deployment.

**Results:**

The observed incidence of LMCA-ASM post-successful PCI was 26.2%, both per patient and per lesion. Lesions with LMCA-ASM had a longer stent diameter, larger stent areas, and larger lumen areas compared to those without LMCA-ASM (4.0 ± 0.5 *vs*. 3.7 ± 0.4 mm, *p* < 0.001; 9.8 ± 2.0 *vs*. 9.0 ± 1.6 ${\mathrm{mm}}^{2}$, *p* < 0.001; 12.3 ± 1.9 *vs*. 10.1 ± 2.1 ${\mathrm{mm}}^{2}$, *p* < 0.001, respectively). The mean external elastic membrane (EEM) area (odds ratio (OR): 1.418 [95% confidence interval (CI): 1.295–1.556]; *p* < 0.001) emerged as an independent predictor of LMCA-ASM. During the observation period, LMCA-ASM did not display any association with device-oriented clinical endpoints (DoCE), which included cardiac death, target vessel-induced myocardial infarction (MI), stent thrombosis, and target lesion revascularization (TLR). Moreover, the DoCE incidence exhibited no significant disparity between patients with or without ASM (13.1 *vs*. 6.0%, *p* = 0.103).

**Conclusions:**

While LMCA-ASM was a not uncommon finding post-PCI, it did not correlate with adverse cardiac events in the present study.

## 1. Introduction

Acute stent mal-apposition (ASM), found on intravascular ultrasound (IVUS) and optical coherence tomography (OCT), frequently denotes incomplete stent apposition to the intimal surface, characterized by some stent struts not being in full contact with the vessel wall following drug-eluting stent (DES) deployment [1, 2, 3]. The origins of ASM within lesions are diverse and multifaceted, stemming either from technical shortcomings (e.g., inadequate stent sizing or suboptimal stent expansion) or vascular morphology and lesion characteristics (e.g., stent placement at bifurcation points, in expansive arteries, in instances of overlapping stents due to extended diffuse lesions, or adjacent to asymmetric calcific plaques) [4, 5]. The design and metal composition of the implanted stent might influence its adaptability to plaque and vessel morphology, thereby dictating the extent of ASM [6]. While the clinical consequences of ASM continue to be debated [7, 8, 9, 10], several studies have associated ASM with an elevated risk of thrombotic events, based on findings associated with stent strut mal-apposition in patients diagnosed with stent thrombosis [9, 10]. The distinct anatomical nuances of the left main coronary artery (LMCA), especially the pronounced mismatch between stent strut and vessel lumen diameters, continue to be hurdles to effective vessel revascularization [11]. However, a comprehensive appraisal of LMCA-ASM is lacking. This study, leveraging IVUS insights, seeks to: (1) ascertain the frequency of IVUS-identified ASM in the LMCA; (2) dissect the morphological characteristics of ASM within the LMCA; (3) pinpoint predictors for LMCA-ASM; and (4) elucidate outcomes associated with IVUS-observed ASM in the LMCA, emphasizing the implications of ASM on stent thrombosis post-IVUS-guided percutaneous coronary intervention (PCI).

## 2. Material and Methods

### 2.1 Study Population

**Fig. 1.**
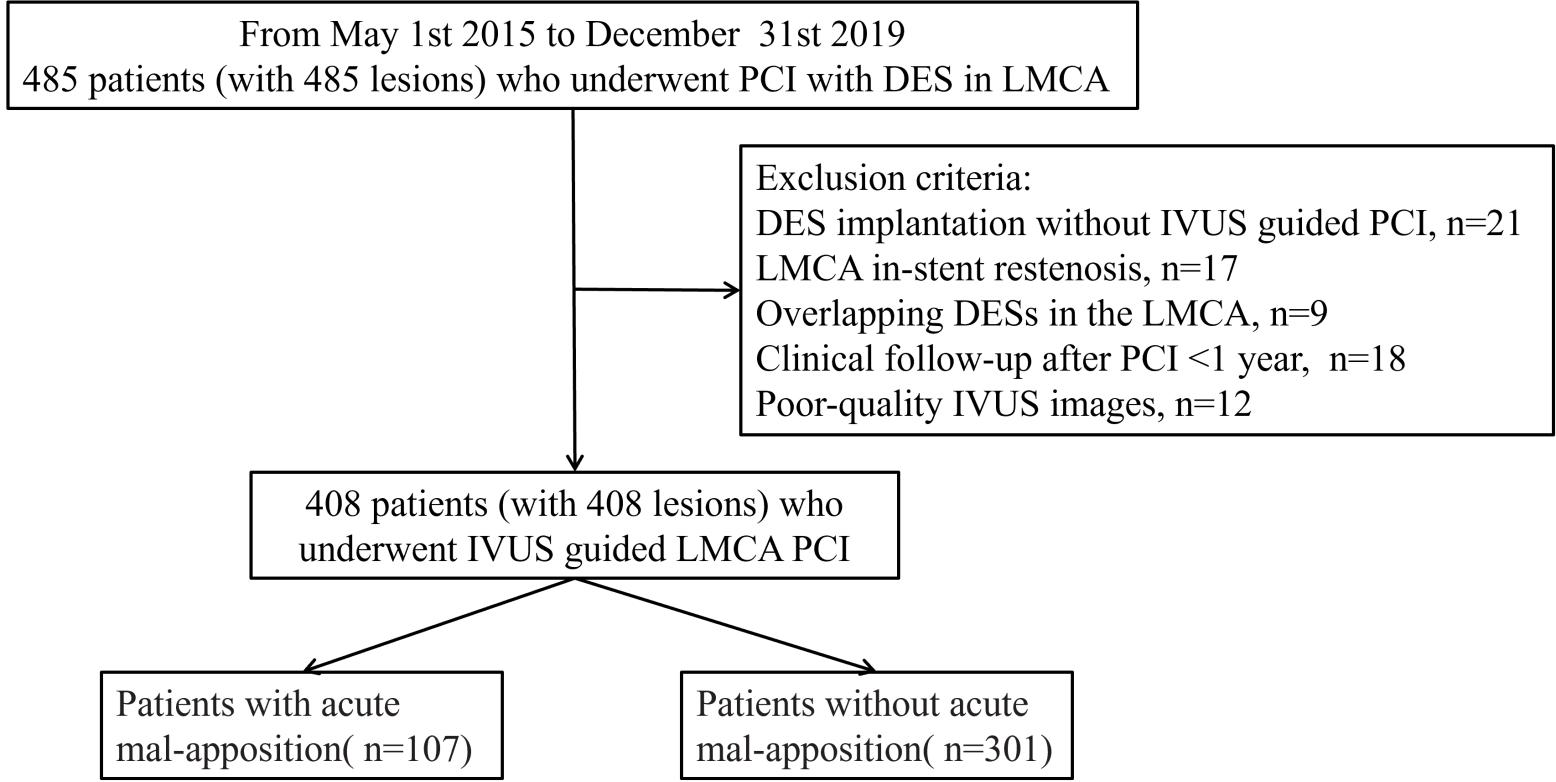
**The study flow chart.** PCI, percutaneous coronary intervention; DES, drug-eluting stent; LMCA, left main coronary artery; IVUS, intravascular ultrasound.

A single-center retrospective assessment was conducted at the Xiangtan Central Hospital, encompassing IVUS imaging of LMCAs between May 1st, 2015, and December 31st, 2019. In 408 patients with 408 lesions, each patient underwent LMCA PCI with a subsequent IVUS examination. Exclusion criteria were: (1) LMCA in-stent restenosis; (2) overlapping DESs within the LMCA; (3) post-PCI clinical follow-up less than a year; and (4) poor-quality IVUS imagery. This study adhered to the 1975 Declaration of Helsinki, and obtained approval from the Ethics Committee of the Xiangtan Central Hospital. Prior to the investigation, written consent was obtained from all participants. Fig. 1 delineates the study flow.

### 2.2 Percutaneous Coronary Intervention

Interventional procedures were performed at the operator’s discretion. Second-generation DESs were used in all cases. Conventional guidelines were employed for all coronary intervention procedures and drug dose standards [12]. Patients were administered an initial dose of 300 mg aspirin and either 300 mg clopidogrel, 180 mg ticagrelor, or 60 mg prasugrel at least 12 hours before the intervention procedure. At the intervention onset, an intravenous heparin bolus of 100 IU/kg was administered, ensuring an activated clotting time between 250 and 300 seconds. Following DES insertion, dual antiplatelet therapy was prescribed for a minimum of 12 months, consisting of 100 mg aspirin along with either 75 mg clopidogrel, 180 mg ticagrelor, or 10 mg prasugrel. During the stent implantation, factors such as the application of mechanical support, the appropriate size of stent and pre- or post-dilation balloon, or concomitant medication, were carried out according to the discretion of the operator. Post-dilation was performed in all stent implantation procedures to rectify the presentation of ASM. In cases with severe calcification or huge vessel size, the operator may have used a larger balloon or higher balloon pressure to correct an unavoidable mal-apposition as far as possible.

### 2.3 Quantitative Coronary Angiography Analysis 

Quantitative coronary angiography (QCA) was performed utilizing QAngio® XA (Medis, Leiden, Netherlands), an offline standard software, and was assessed by three evaluators uninformed of the study’s objectives. The QCA evaluation encompassed metrics such as minimal lumen diameter, diameter stenosis both pre- and post-PCI, lesion length, referential vessel diameter, and calcification presence [13].

### 2.4 IVUS Image Analysis 

**Fig. 2.**
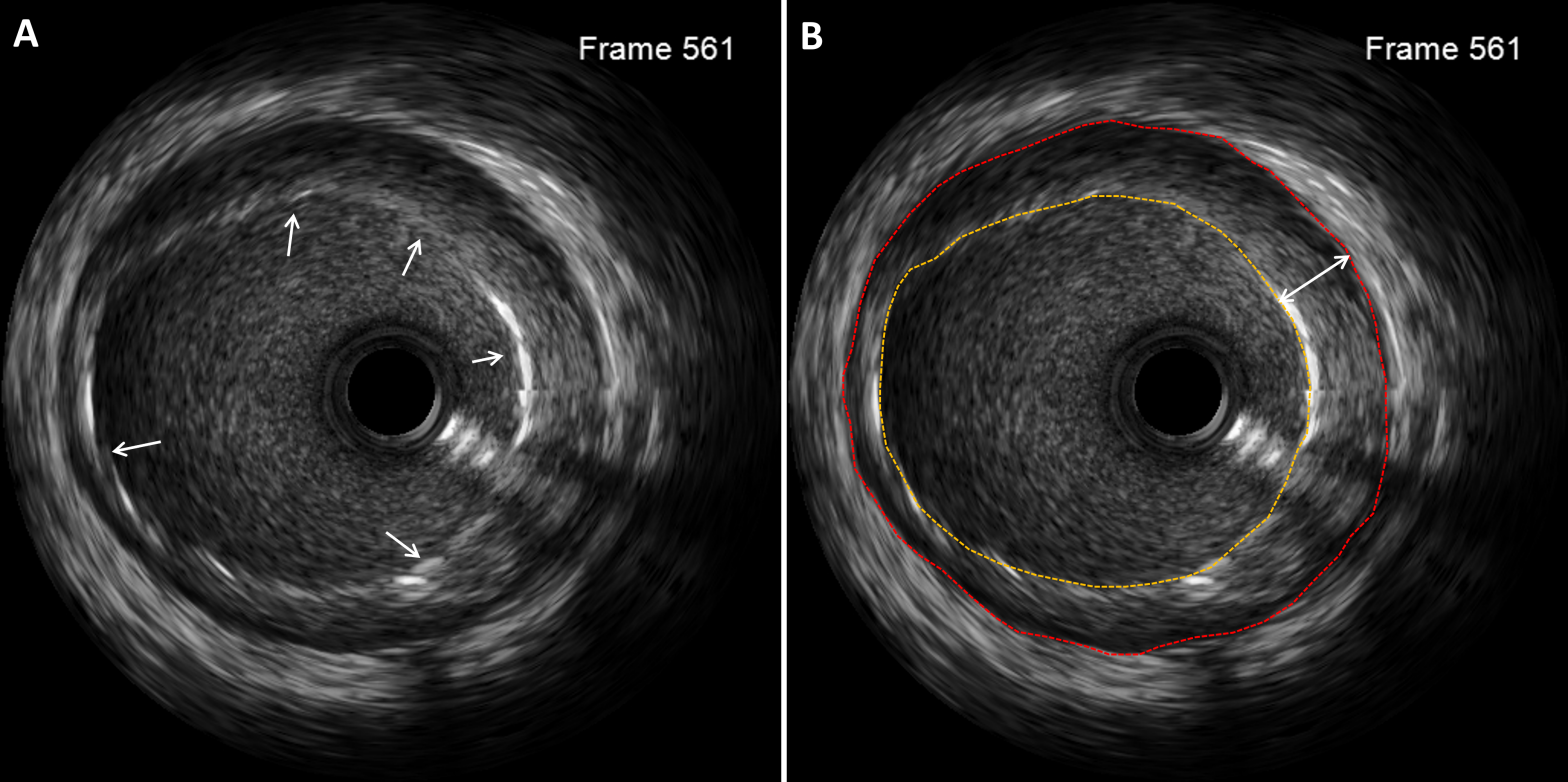
**Representative images of acute stent mal-apposition detected on intravascular ultrasound.** (A) Stent mal-apposition (white arrows). (B) Mal-apposition area was calculated by subtracting the stent area from the lumen area. Lumen area (red broken line), stent area (yellow broken line) and maximal depth (white two-way arrow) of mal-apposition.

Both pre- and post-PCI IVUS imaging employed a 40-MHz IVUS system (${\mathrm{OptiCross}}^{\text{TM}}$, Boston Scientific, Marlborough, MA, USA) with an automated pullback at 0.5 mm/s following intracoronary administration of nitroglycerin (0.1–0.2 mg). The IVUS catheter was extended more than 10 mm past the stent into the distal segment and positioned more than 10 mm in front of the stent. The evaluation of images was conducted by evaluators who were blinded to the patient and their interventional procedure information, utilizing dedicated offline software (QIvus®, Medis, Leiden, Netherlands). Stent mal-apposition was characterized by stent struts discernibly detached from the adjoining intima vessel wall, with blood speckles behind the strut, excluding strut overlap with a side branch [13] (Fig. 2). Metrics such as location, distance, and length per cross-sectional area (CSA) in mal-apposed segments were delineated and evaluated. The captured metrics included the in-stent CSA, minimum lumen and stent areas in lesion segments, external elastic membrane (EEM), lumen, plaque + media, stent area, and plaque burden.

### 2.5 Clinical Follow-up and Outcomes

Device-oriented clinical endpoints (DoCE) included cardiac death, target vessel-induced myocardial infarction (MI), stent thrombosis, and target lesion revascularization (TLR), as defined per the Academic Research Consortium standards [14]. Six-month clinical reviews were undertaken either in person or via telecommunication. Study participants were followed up for a minimum of one year.

### 2.6 Statistical Analysis

Data representation for continuous variables employed the mean ± standard deviation, or median (interquartile range), while categorical variables were denoted using a number (percentage). Continuous data comparisons utilized the Student’s *t*-test or the Mann-Whitney U test, and categorical data were assessed using the Fisher’s exact or chi-square tests. Determinants of ASM predictors were reported via odds ratio (OR) with 95% confidence interval (CI) in both univariate and multivariate logistic regression. In univariate analyses, each factor was examined one by one to assess its association with the outcome variable. We then selected those with significant associations with outcomes (*p* < 0.05), which were further analyzed. In multivariable analyses, a stepwise approach was used and a prespecified set of factors referred to previous studies. The selected factors were entered together in a Cox regression model, and their adjusted hazard ratio (HR) and 95% CI were estimated. This adjustment process takes into account the interaction between factors. The validity of the Cox proportional hazards model was assessed by testing the proportional hazards assumption by plotting residual plots of covariates over time. Kaplan-Meier curves were employed for clinical event rate estimations, and compared using the log-rank test. In scenarios of multiple DoCE per patient, only the initial event per patient was considered. Significance was set at *p* < 0.05. SPSS 26.0 (SPSS Inc., Chicago, IL, USA) in all analytical procedures.

## 3. Results

The study encompassed 408 patients with 408 LMCA lesions, all of whom underwent an IVUS assessment immediately following successful DES deployment. Dissecting the lesions bearing ASM, the predominant ASM location was the proximal LMCA at 64.5% (69 of 107), followed by the LMCA body at 26.2% (28 of 107), and the distal LMCA at 9.3% (10 of 107). In lesions with an ASM, the predominant ASM location was the proximal LMCA in 64.5% (69 of 107), followed by the LMCA body at 26.2% (28 of 107), and the distal LMCA at 9.3% (10 of 107). For ASM diagnosis, both intra- and inter-observer variations showed consistent results (Cohen’s kappa values at 0.92 and 0.89, respectively).

### 3.1 Baseline Clinical Characteristics

Baseline characteristics showed no difference between the patients with ASM versus those without ASM (Table 1).

**Table 1.** **Clinical characteristics**.

		Patients with acute mal-apposition	Patients without acute mal-apposition	*p* value
		(n = 107)	(n = 301)	
Age (year)		61.2 ± 11.8	62.0 ± 10.9	0.477
Males, n (%)		81 (75.7)	213 (70.8)	0.328
Current smoker, n (%)		32 (29.9)	94 (31.2)	0.799
Diabetes mellitus, n (%)		29 (27.1)	75 (24.9)	0.656
Hypertension, n (%)		86 (80.4)	232 (77.1)	0.480
Hyperlipidemia, n (%)		70 (65.4)	207 (68.8)	0.524
Chronic kidney disease, n (%)		18 (16.8)	54 (17.9)	0.794
Body mass index, kg/$m^{2}$		28.5 ± 5.3	29.0 ± 5.4	0.347
Previous MI, n (%)		30 (28.3)	78 (25.9)	0.669
Previous PCI, n (%)		34 (31.8)	99 (32.9)	0.833
Clinical presentation				
	ST-segment elevation MI, n (%)	12 (11.2)	33 (11.0)	0.943
	Non ST-segment elevation MI, n (%)	19 (17.8)	43 (14.3)	0.390
	Unstable angina, n (%)	24 (22.4)	82 (27.2)	0.330
	Stable coronary artery disease, n (%)	52 (48.6)	135 (44.9)	0.504
Laboratory data				
	Hemoglobin, g/dL	14.2 ± 1.6	14.2 ± 2.2	0.949
	White blood cell count (×${\mathrm{10}}^{3}$/µL)	8.17 ± 2.69	7.67 ± 2.80	0.102
	Creatinine (mg/dL)	1.3 ± 0.9	1.1 ± 0.7	0.013
	Total cholesterol (mg/dL)	168.6 ± 66	163.7 ± 67.4	0.507
	Triglyceride (mg/dL)	140.6 ± 130.1	138.3 ± 103.8	0.854
	HDL-cholesterol (mg/dL)	41.1 ± 13.9	39.0 ± 15.6	0.198
	LDL-cholesterol (mg/dL)	110.8 ± 48.5	104.4 ± 53.4	0.276
	Peak CK-MB (ng/mL)	42.7 ± 7.5	42.9 ± 7.7	0.816
	hs-CRP (mg/dL)	4.7 ± 1.8	4.8 ± 2.3	0.684
Drug				
	Aspirin, n (%)	107 (100)	301 (100)	-
	DAPT, n (%)	107 (100)	301 (100)	-
	Beta-blocker, n (%)	57 (53.3)	147 (48.8)	0.431
	ACE inhibitor/ARB, n (%)	54 (50.5)	144 (47.8)	0.641
	Statin, n (%)	102 (95.3)	292 (97.0)	0.411
	Calcium channel blocker, n (%)	29 (27.1)	81 (26.9)	0.969

Values are mean ± SD or n (%). ACE, angiotensin-converting enzyme; ARB, angiotensin II receptor blocker; DAPT, dual-antiplatelet therapy; HDL, high density lipoprotein; LDL, low-density lipoprotein; MI, myocardial infarction; PCI, percutaneous coronary intervention; hs-CRP, high-sensitivity C-reactive protein; CK-MB, creatine kinase MB.

### 3.2 Angiographic and Procedural Findings

Table 2 indicates both QCA outcomes and procedural results. Lesions within the ASM group displayed a more expansive minimal lumen diameter (3.2 ± 1.1 *vs*. 3.0 ± 0.9 mm, *p* = 0.044) and a superior reference vessel diameter (4.4 ± 0.4 *vs*. 3.6 ± 0.5 mm, *p* < 0.001). ASM lesions frequently employed more extended stents (15.3 ± 7.0 *vs*. 9.4 ± 6.2 mm, *p* < 0.001) in comparison with non-ASM lesions.

**Table 2.** **Angiographic and procedural findings**.

		Lesions with acute mal-apposition	Lesions without acute mal-apposition	*p* value
		(n = 107)	(n = 301)	
Lesion length, mm		11.3 ± 6.7	12.4 ± 6.2	0.112
Minimal lumen diameter, mm		3.2 ± 1.1	3.0 ± 0.9	0.044
% of stenosis		42.0 ± 16.8	39.6 ± 12.4	0.119
Reference vessel diameter, mm		4.4 ± 0.4	3.6 ± 0.5	0.000
Stent diameter, mm		3.4 ± 0.3	3.4 ± 0.4	0.616
Total stent length, mm		15.3 ± 7.0	9.4 ± 6.2	0.000
Adjuvant procedure				
	Adjuvant dilatation	107 (100)	301 (100)	1
	Adjuvant balloon diameter, mm	3.61 ± 0.63	3.5 ± 0.56	0.092
	Adjuvant balloon length, mm	8.14 ± 2.39	8.23 ± 2.99	0.755
	Maximum balloon pressure, atm, median (interquartile range)	18.4 (18.2–18.6)	18.4 (18.2–18.5)	0.805

Values are mean ± SD or n (%) or median (interquartile range).

### 3.3 IVUS Findings

Table 3 delineates postprocedural IVUS outcomes. Longitudinal ASM dimensions were recorded at 1.0 ± 0.2 mm, with a maximum ASM area of 3.2 ± 0.9 ${\mathrm{mm}}^{2}$, a total mal-apposed volume of 6.5 ± 2.4 ${\mathrm{mm}}^{3}$, and a maximal strut-to-vessel wall distance of 1.8 ± 0.4 mm. Few stents (18 out of 408) met the standard criteria for under-expansion (MSA <8 ${\mathrm{mm}}^{2}$). Lesions with ASM exhibited a longer stent diameter, larger stent areas, and larger lumen areas in contrast to those devoid of ASM. Yet, tissue protrusion prevalence remained consistent across both cohorts (34.6 *vs*. 33.6%, *p* = 0.847). A univariate logistic assessment revealed associations between minimal lumen diameter, adjuvant balloon diameter, and mean EEM area with LMCA-ASM (Table 4). Subsequent multivariate logistic analysis found a mean EEM area (OR 1.419; 95% CI 1.295–1.556; *p* < 0.001) to be an independent predictor for LMCA-ASM (Table 5).

**Table 3.** **IVUS findings**.

	Lesions with acute mal-apposition	Lesions without acute mal-apposition	*p* value
	(n = 107)	(n = 301)	
Maximal strut-to-vessel wall distance, mm	1.8 ± 0.4	-	-
Mal-apposition length, mm	1.0 ± 0.2	-	-
Total mal-apposed volume, ${\mathrm{mm}}^{3}$	6.5 ± 2.4	-	-
Maximum mal-apposition area, ${\mathrm{mm}}^{2}$	3.2 ± 0.9	-	-
Tissue protrusion	37 (34.6)	101 (33.6)	0.847
Minimal stent diameter, mm	3.7 ± 0.4	3.5 ± 0.4	0.000
Maximal stent diameter, mm	4.0 ± 0.5	3.7 ± 0.4	0.000
Minimal stent area, ${\mathrm{mm}}^{2}$	9.8 ± 2.0	9.0 ± 1.6	0.000
Minimum lumen area, ${\mathrm{mm}}^{2}$	9.9 ± 2.1	8.9 ± 1.7	0.000
Mean lumen area, ${\mathrm{mm}}^{3}$/mm	12.3 ± 1.9	10.1 ± 2.1	0.000
Mean stent area, ${\mathrm{mm}}^{3}$/mm	11.5 ± 2.0	9.8 ± 1.8	0.000
Mean EEM area, ${\mathrm{mm}}^{3}$/mm	18.4 ± 3.2	15.6 ± 2.7	0.000
Mean plaque area, ${\mathrm{mm}}^{3}$/mm	7.9 ± 2.3	7.0 ± 2.6	0.001

EEM, external elastic membrane; IVUS, intravascular ultrasound.

**Table 4.** **Univariate analysis for predictors of acute mal-apposition**.

	OR	95% CI	*p* value
Age	0.993	0.973–1.013	0.476
Male sex	0.777	0.468–1.290	0.329
Diabetes mellitus	1.120	0.680–1.847	0.656
Hypertension	1.218	0.704–2.106	0.480
Hyperlipidemia	0.859	0.524–0.859	0.524
Chronic kidney disease	0.925	0.515–1.662	0.795
Previous MI	1.114	0.679–1.826	0.669
Unstable angina	0.772	0.459–1.299	0.330
LDL-cholesterol	1.002	0.998–1.007	0.275
Peak CK-MB	0.997	0.968–1.026	0.817
Lesion length	0.968	0.930–1.008	0.112
Minimal lumen diameter	1.324	1.042–1.682	0.022
Maximum balloon pressure	0.759	0.253–2.284	0.624
Adjuvant balloon diameter	1.404	0.980–2.011	0.092
Mean EEM area	1.418	1.271–1.575	0.000

OR, odds ratio; CI, confidence interval; MI, myocardial infarction; LDL, low-density lipoprotein; CK-MB, creatine kinase MB; EEM, external elastic membrane.

**Table 5.** **Multivariate analysis for predictors of acute mal-apposition**.

	OR	95% CI	*p* value
Minimal lumen diameter	1.277	0.979–1.665	0.071
Adjuvant balloon diameter	1.518	0.988–2.333	0.057
Mean EEM area	1.419	1.295–1.556	0.000

OR, odds ratio; CI, confidence interval; EEM, external elastic membrane.

### 3.4 Clinical Outcomes

**Fig. 3.**
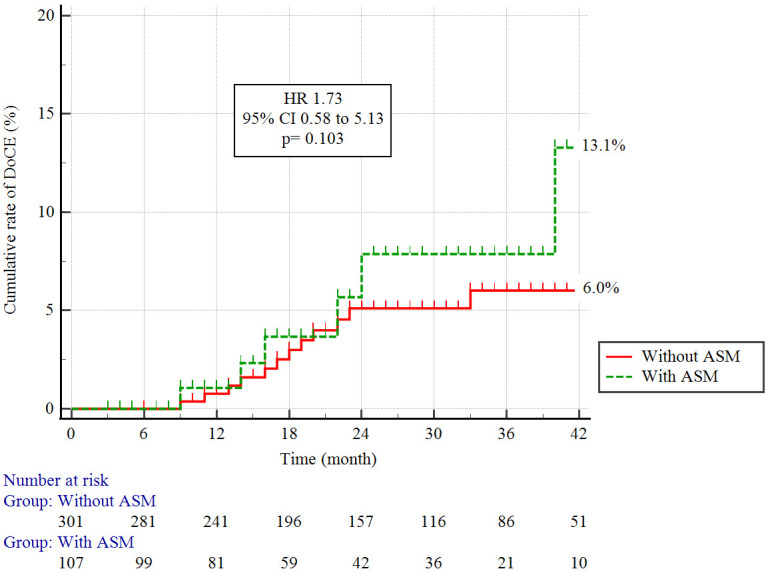
**Kaplan–Meier cumulative incidence curves for DoCE.** DoCE, device-oriented clinical endpoint; HR, hazard ratio; CI, confidence interval; ASM, acute stent mal-apposition.

The follow-up period was 25.3 ± 13.4 months. Clinical events are presented in Table 6. Fig. 3 presents the Kaplan-Meier curves of the DoCE in patients with LMCA-ASM versus those without LMCA-ASM. Metrics such as cardiac mortality, target vessel-induced MI, stent thrombosis, and TLR were similar between patients with LMCA-ASM versus those without LMCA-ASM. Notably, even with ASM inclusion in both univariate and multivariate evaluations, ASM did not emerge as an independent predictor for DoCE (Table 7).

**Table 6.** Clinical outcomes*.

	Lesions with acute mal-apposition	Lesions without acute mal-apposition	*p* value
	(n = 107)	(n = 301)	
DoCE, n (%)	14 (13.1)	18 (6.0)	0.103
Cardiac death, n (%)	2 (1.9)	2 (0.7)	0.866
Target vessel-related myocardial infarction, n (%)	8 (7.5)	13 (4.3)	0.501
Stent thrombosis, n (%)	2 (1.9)	0 (0)	0.769
Target lesion revascularization, n (%)	7 (6.5)	12 (4.0)	0.643

*Data are expressed as number of events (cumulative 3.5-year rate of event). DoCE, Device-oriented clinical endpoints.

**Table 7.** **Univariate analysis for predictors of DoCE**.

	HR	95% CI	*p* value
Age	1.007	0.985–1.030	0.532
Male sex	1.111	0.656–1.882	0.695
Unstable angina	0.989	0.569–1.721	0.970
Previous MI	1.013	0.582–1.764	0.962
Body mass index	1.019	0.974–1.066	0.424
Diabetes mellitus	0.889	0.512–1.546	0.678
Hypertension	1.619	0.846–3.097	0.146
Current smoker	0.749	0.442–1.270	0.283
Peak CK-MB	1.009	0.979–1.041	0.557
LDL-cholesterol	1.001	0.997–1.006	0.652
Beta-blocker	1.612	0.979–2.654	0.061
Statin	0.678	0.212–2.168	0.513
Minimal stent area	0.928	0.803–1.072	0.310
Minimal lumen diameter	0.848	0.652–1.103	0.218
Stent diameter	0.980	0.535–1.796	0.948
Tissue protrusion	0.941	0.560–1.580	0.817
Total mal-apposition volume	1.096	0.818–1.469	0.538
Maximum balloon pressure	0.860	0.249–2.965	0.811
Adjuvant balloon diameter	1.119	0.759–1.649	0.571
Mean EEM area	1.027	0.937–1.125	0.566

HR, hazard ratio; MI, myocardial infarction; CK-MB, creatine kinase MB; LDL, low-density lipoprotein; EEM, external elastic membrane; DoCE, Device-oriented clinical endpoints; CI, confidence interval.

## 4. Discussion

Utilizing IVUS data, this investigation represents an effort to elucidate the clinical consequences of residual ASM in the LMCA. The salient outcomes of this study underscore several aspects: (1) The incidence of post-stent LMCA-ASM prevalence was 26.2%, both on a per-patient and per-lesion basis, as determined by IVUS; (2) Compared to lesions without LMCA-ASM, lesions with LMCA-ASM had a longer stent diameter, larger stent areas, and larger lumen areas, with the mean EEM area emerging as an independent predictor; (3) The observational period revealed no association between LMCA-ASM and DoCE; (4) The incidence of DoCE shows no significant difference in the presence or absence of ASM.

### 4.1 Prevalence of ASM

ASM represents a scenario wherein the stent struts fail to make intimate contact with the vessel wall’s intimal layer after primary stent deployment [2]. Mal-apposition of stent struts has been documented through both IVUS and OCT techniques. In our cohort, the incidence of IVUS-detected LMCA-ASM was 26.2% (107 out of 408 lesions). Among various studies, the IVUS-detected fraction of acutely mal-apposed struts has been an average of 13% post-stent implementation (ranging from 7.2% to 38.5%). Steinberg *et al*. [15] evaluated 1580 patients from the IVUS sub-studies of multiple TAXUS trials and found the ASM prevalence fluctuations between 7.2% and 9.7% for both bare metal stents (BMS) and TAXUS drug-eluting stents. In the IVUS sub-cohort of the Harmonizing Outcomes with Revascularization and Stents in Acute Myocardial Infarction (HORIZONS-AMI) trial [16], ASM rates were 34.3% for paclitaxel-eluting stents and 40.3% for BMS across 263 native coronary lesion cases that demonstrated an ST segment elevation myocardial infarction (STEMI). In another study in STEMI patients, the incidence of ASM was 33.8% and 38.5% for BMS and sirolimus-eluting stent (SES), respectively [17]. Wang *et al*. [18], in the encompassing ADAPT-DES (Assessment of Dual Antiplatelet Therapy With Drug-Eluting Stents) study, found the incidence of ASM was 14.4% per patient and 12.6% per lesion post-DES implantation as identified by IVUS. These findings are consistent with the prevalence of ASM reported in our analysis.

IVUS might underrepresent the actual occurrence of ASM. OCT, possessing superior resolution compared to IVUS, offers enhanced accuracy in discerning minuscule strut mal-appositions. Post-procedural ASM, as detected by OCT, averages around 51% after stent placements, ranging between 39.1% and 72.3% [5, 8, 19, 20, 21]. This indicates pervasive ASM post-implantation when adopting highly sensitive assessment methodologies.

### 4.2 Risk Factors for ASM

The possible risk factors for ASM are multifaceted and varied, encompassing technical procedural elements such as stent type, stent under-sizing, or under-expansion, and inherent vessel characteristics such as positive vessel remodeling, thrombus dissolution post-stenting, or suboptimal neointimal restoration following intimal damage. Lesion morphologies, including bifurcation zone stenting, large vessel diameter interventions, long diffuse lesion treatment necessitating overlapping stents, or stenting across eccentric calcified plaques or nodules, also play a role [1, 5]. In our research, due to the expansive mean EEM area, a pronounced ASM prevalence was observed in the left main coronary artery. In line with the demonstration from a previous study that larger vessels may be predictors of ASM [15], these findings revealed higher frequency of ASM with increasing lesion complexity and morphological abnormality. Agrawal *et al*. [22], in a comprehensive analysis, demonstrated localized vessel diameter expansion, recognized as positive remodeling or the “Glagov effect” [23], as the predominant factor in strut mal-apposition, found in 74% of stents exhibiting mal-apposition. Im *et al*. [4], analyzed 351 patients with 356 lesions undergoing OCT examinations during PCI and determined that severe diameter stenosis, the presence of calcified lesions, and extended stent lengths were closely linked with OCT-identified ASM. Kubo *et al*. [24] found a greater ASM prevalence in patients with unstable angina (67%) than in their stable counterparts (33%) during PCI. Lesions with an angle of ≥45° on angiography, examined by Minami *et al*. [25], manifested an increased incidence of ASM after second-generation DES implantation. From a theoretical standpoint, the augmentation of the risk of stent thrombosis may be attributable to exposed stent struts and localized flow anomalies induced by ASM. Qu *et al*. [26], utilizing computational simulation models, deduced that stent thrombogenicity increased significantly as the ASM gap distance widened to 150 µm, but this plateaued beyond this threshold.

### 4.3 Outcomes Associated with ASM

While both IVUS and OCT investigations have identified stent under-expansion as a pivotal independent predictor of stent-associated outcomes, the consequential effects of ASM on clinical adverse outcomes, specifically in-stent restenosis and stent thrombosis, remain uncertain [27]. *In vitro* analyses [26], coupled with intravascular imaging examinations [9, 28], suggest the theoretical association between exposed mal-apposed struts and an elevated likelihood for localized thrombus development, potentially due to induced flow disruptions and protracted healing. Nevertheless, across varied studies involving different patient demographics, stent categories, and imaging modalities, the results found no correlation between ASM and unfavorable cardiac incidents following DES placement, similar to the results from our study [5, 7, 8, 16, 18, 21, 29]. The IVUS-oriented sub-analysis of 2072 patients encompassing 2446 lesions from the ADAPT-DES study revealed no discernible link between ASM and major adverse cardiovascular events (MACE) during a 2-year post-intervention observation [18]. Presently, there is no universally accepted criteria to assess ASM severity linked to adverse cardiovascular events. European expert consensus suggests that pronounced ASM conditions characterized by stent mal-apposed depths exceeding 400 µm or stent mal-apposed lengths ≥1 mm warrant intervention to preclude potential late stent thrombosis post-DES implementation [30]. A recent aggregation of OCT data from six randomized trials found that ASM, as delineated by the European consensus, did not influence the risk for 5-year cardiac adverse events [21].

Stent mal-apposition is separate from stent under-expansion. The CLI-OPCI II sub-analysis evaluated OCT outcomes across 1002 lesions in 832 patients and found an association between stent under-expansion and MACE but no such correlation with strut mal-apposition [7]. Notably, evaluations defining the clinical ramifications of ASM primarily categorized mal-apposition in binary terms existent or non-existent and found no significant association with adverse outcomes. Specific investigations assessed the severity of strut mal-apposition by considering metrics such as distance from the vessel wall or dimensions of the mal-apposed segment, but no adverse correlations were demonstrated [7].

The location of ASM is important. ASM occurrence is predominantly observed at the stent boundary [4], especially when in a tapered vessel like the left anterior descending coronary artery. Persisting stent mal-apposition in the case of complex bifurcation lesions can lead to the guidewire navigating through stent struts in subsequent distal lesion procedures [31]. Successful guidewire insertion within the stent doesn’t negate the risk; catheter tips from balloons, stents, or intravascular imaging tools could become ensnared at the stent’s proximal end, potentially triggering longitudinal stent deformation [32].

### 4.4 Study Limitations

First, this research, being non-randomized and observational, draws from a single center’s low event occurrences, with a limited sample size, and a brief observational period, thus increasing the possibility of potential selection bias. Second, the retrospective approach to clinical outcome data extraction possibly underlines the reporting of deficiencies. Third, the older version of the dataset used in this study may contribute to its under-representation of data and events since data recorded manually may introduce potential errors. Finally, the resolution of the IVUS images used was relatively lower than OCT; the present study has a lack of agreement of quantitative definition of acute stent mal-apposition.

## 5. Conclusions

Post-PCI, LMCA-ASM findings were not uncommon but lacked an association with adverse cardiac events in this investigation. Future studies with a larger sample size and a longer follow-up will be pivotal in determining the associations between adverse clinical events and LCMA-ASMs.

## Data Availability

The datasets generated during and/or analyzed during the current study are available from the corresponding author on reasonable request.
